# Single and consecutive 10-day remote ischemic preconditioning modify physical performance, post-exercise exerkine levels, and inflammation

**DOI:** 10.3389/fphys.2024.1428404

**Published:** 2024-11-11

**Authors:** Magdalena Kochanowicz, Paulina Brzezinska, Jan Mieszkowski, Andrzej Kochanowicz, Bartlomiej Niespodzinski, Marcin Surmiak, Joanna Reczkowicz, Andzelika Borkowska, Jedrzej Antosiewicz

**Affiliations:** ^1^ Department of Physiotherapy, Medical University of Gdansk, Gdańsk, Poland; ^2^ Department of Gymnastics and Dance, Gdansk University of Physical Education and Sport, Gdańsk, Poland; ^3^ Department of Biological Foundations of Physical Education, Faculty of Health Sciences and Physical Education, Kazimierz Wielki University, Bydgoszcz, Poland; ^4^ Center for the Development of Therapies for Civilization and Age-Related Diseases, Jagiellonian University Medical College, Kraków, Poland; ^5^ Department of Internal Medicine, Faculty of Medicine, Jagiellonian University Medical College, Kraków, Poland; ^6^ Department of Bioenergetics and Physiology of Exercise, Medical University of Gdańsk, Gdańsk, Poland

**Keywords:** endurance exercise, ischemic preconditioning (IPC), skeletal muscle damage, inflammation, wingate anaerobic performance

## Abstract

**Purpose:**

Remote ischemic preconditioning (RIPC) is a method of protection against induced ischemia reperfusion injury, and an increasing number of studies showed some of its inconclusive ergogenic effects in sports. RIPC involves short cycles of cuff inflation followed by its deflation which may affect many body systems. While most of the studies focus on single RIPC effects, there is insufficient data regarding training-like repeated RIPC interventions. Thus, in this study, we analyzed the effect of a single- and consecutive 10-day RIPC procedure on a single leg, focusing on the exerkine levels and changes in inflammation markers following the Wingate Anaerobic Test (WAnT).

**Methods:**

Two single-blinded, sham-controlled protocols were designed to evaluate the 1) single (crossover study) and 2) consecutive 10-day (parallel study) RIPC effects on the WAnT performance and exercise-induced lactate, glucose, exerkine, and inflammation markers (BDNF; IL-6; IL-10; IL-15; LIF; oncostatin M). In each protocol, 37 physically active men (19.98 ± 1.17 years) were randomly assigned into two groups according to a particular study design.

**Results:**

An increase in participants’ mean (4.81%, p < 0.05) and peak power (6.25%, p < 0.05) during the WAnT was observed only after the consecutive 10-day RIPC. Similarly, a significant 15.5% (p < 0.05) decrease in the IL-6 concentration 120 min after the WAnT was observed only in the consecutive 10-day RIPC protocol, as well as a 12.2% (p < 0.01) increase in oncostatin M 60 min after the WAnT.

**Conclusion:**

The results demonstrate the efficacy of the consecutive 10-day RIPC procedure in modulating exercise performance and post-exercise inflammation markers.

## Introduction

A growing number of sports and medicine studies indicate the importance of searching for a new protective method against induced ischemia-reperfusion injury (Mieszkowski et al., 2021). Increasing focus is being placed on ischemic preconditioning (IPC), a procedure known to induce a protective response in internal organs and tissues against ischemic damage (Murry et al., 1986; Tapuria et al., 2008; Bailey et al., 2012). Taking into account the nature of this procedure and its similarities with exercises (tissue blood flow changes during exercises), the development of tissue resistance under ischemic conditions may affects sport performance and muscle activity (Mieszkowski et al., 2019; Niespodziński et al., 2021). Typically, the IPC procedure relies on brief cycles of limb ischemia and reperfusion-induced, typically by inflating and deflating a blood pressure cuff. As a tissue response, it can induce local (IPC, tissue exposed to ischemia) and distal [remote IPC (RIPC), other tissues through blood flow] changes and higher resistance of tissues undergoing preconditioning for cycles of ischemia (Mieszkowski et al., 2021). Because of the intermittent nature of blood flow and oxygen supply during intense muscle actions, it was proposed to employ IPC prior to physical exercise, with results showing increased muscle force output (Libonati et al., 1998; Paixão et al., 2014).

Moreover, the benefits of RIPC have been observed in submaximal (Mieszkowski et al., 2019) and graded maximal incremental exercise on a cycle ergometer (de Groot et al., 2010) in submaximal and maximal swim tests (Jean-St-Michel et al., 2011), graded maximal treadmill running tests (Bailey et al., 2012), and local neuromuscular performance (Niespodziński et al., 2021). Most findings have confirmed the advantages of RIPC in enhancing athletic performance, and RIPC has been postulated as a natural ergogenic aid (Paixão et al., 2014; Gibson et al., 2015). Many of the related research interventions focus mainly on single RIPC procedures and their effects on sports results and physical performance (de Groot et al., 2010; Crisafulli et al., 2011; Patterson et al., 2015). Currently, there is limited knowledge regarding the application of the RIPC procedure as a repetitive training method during normal sports training sessions over several days (Kimura et al., 2007; Foster et al., 2011; Mieszkowski et al., 2019; Niespodziński et al., 2021). This approach may trigger an adaptative response in the muscle, enhancing its resilience to a stress condition (Mieszkowski et al., 2021). Recent studies have suggested that myokines may play a pivotal role in mediating the protective effects of ischemic preconditioning. In animal models, IPC has been shown to stimulate the release of interleukin 10 (IL-10) and stromal-derived factor-1 (SDF-1 or CXCL12), which in turn can help reduce inflammation, promote angiogenesis, and enhance the survival of cells (Cai et al., 2013; Davidson et al., 2013). While the exact mechanisms by which myokines exert their protective effects are still being investigated, these findings suggest that ischemic preconditioning may represent a promising method affecting the body’s functioning. Many authors suggest that IPC applied on skeletal muscles intensifies their endocrine function by increasing synthesis and release a number of proteins, cytokines, and low-molecular weight compounds which enter the systemic circulation and, with the blood flow, affect not only muscles but many other tissues (Kido et al., 2015; Schnyder and Handschin, 2015; Mieszkowski et al., 2021).

Considering the current lack of knowledge about the specific of a muscle tissue’s secretion regulation, depending on the effect of the ischemic preconditioning procedure, it seems essential to comprehensively explore the mechanism involved in the body’s response to this type of training.

Moreover, gaining a deeper understanding of tissue-specific secretion responses to both single-day and consecutive 10-day RIPC procedures, particularly in relation to physical performance, could provide a valuable foundation for future research. Such studies are relevant not only in the realm of sports and physical performance but also hold significant potential in medical and health science applications.

## Materials and methods

### Ethics statement

The study was conducted ethically, with the Bioethics Committee for Clinical Research reviewing the protocol and ensuring compliance with ethical principles (positive decision number KB-24/16). Participants were fully informed of the study procedures and their right to withdraw at any time. The decision to keep participants unaware of the study’s objectives was made to prevent biases. The ethical approval and informed consent process respected the participants’ autonomy and dignity and was conducted according to the Declaration of Helsinki.

### Experimental overview

The study involved two single-blinded, sham-controlled experimentally designed protocols to assess the single RIPC (Protocol - A) and consecutive 10-day RIPC (Protocol - B) effects on maximal anaerobic performance and post-exercise changes in inflammation markers.

All participants were assigned to complete both protocols. The participants were unaware of the group assignments and the differences in the procedures. The study started with Protocol – A and the protocol - B was performed 1 week later.

**FIGURE 1:**
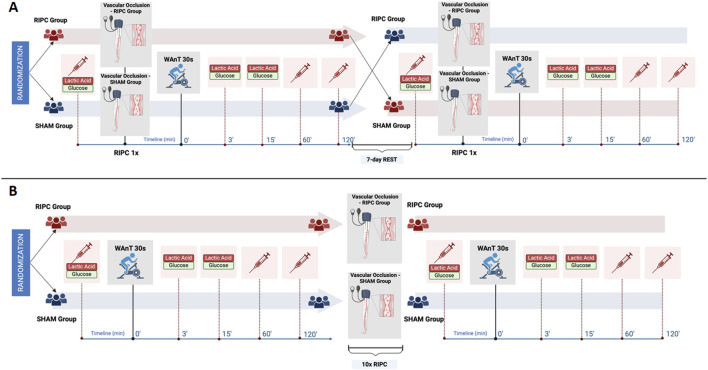
Experimental overview of **(A)** single remote ischemic preconditioning (RIPC) protocol and **(B)** consecutive 10-day RIPC intervention protocol. Note: WAnT – Wingate Anaerobic Test. The figure was created under BioRender license.

On the first day of each protocol, in the early morning hours (between 8:00 - 10:00 a.m.) measurements of the participants’ basic anthropometric characteristics were done. In addition, in protocol – B, measurements were repeated after 10-day of interventions. The week before the start of the experiment, participants attended a familiarization session in order to acquaint themselves with the research protocols and planned experiment. The research protocols are depicted in detail in Figure 1.

#### Protocol - A. single RIPC intervention

In this crossover-controlled trial, all participants were randomly assigned to one of two study groups. One group followed a single RIPC procedure (Group I), while the other group followed a sham-controlled procedure (Group II). Immediately after the interventions, both groups performed the maximal anaerobic effort in the form of the Wingate Anaerobic Test (WAnT). For serum analysis, blood samples were collected immediately before the intervention as well as at 60 min and 120 min post WAnT. Additionally, blood samples for measuring participants’ blood lactate and glucose levels were taken immediately before the intervention and then again at 3 and 15 min after completing the WAnT. After 7 days of rest, the participants switched groups and performed analogous research procedures according to the study design (Figure 1A).

#### Protocol - B. consecutive 10-day RIPC intervention

In this parallel study design, all participants were randomly assigned to one of two study groups, in which one group received consecutive 10-day RIPC and the other 10-day SHAM intervention. Before the start and on the morning following the last day of the 10-day intervention, participants performed WAnT, during which blood samples were taken at the same time points as in Protocol – A of the study (Figure 1B).

### Participants

Forty healthy, physically active men not training or competing professionally in any sports discipline (19.98 ± 1.17 years of age) participated in the study. None of the participants had any history of structured sports training, and they were recruited through an open advertisement and letters of intent. None of the participants suffered from neuromuscular, nervous system, or cardiovascular system diseases, cancers, hyperparathyroidism, or mental disorders. Furthermore, they had a negative history of taking any medications or drugs (up to 6 months prior to the study) that could significantly affect their sports performance and the research tasks of the present study.

Out of the recruited participants, 37 were included in the analysis. Two were excluded due to upper respiratory tract infections during the study, and one was excluded due to a wrist injury that prevented the individual from performing the exercise tests. The participants were randomly assigned to one of two study groups in each of the protocols.

Table 1 shows the basic anthropometric characteristics of the groups in protocol A. Table 2 shows the characteristics of the group in protocol B, which comprises the same pool of participants.

**TABLE 1:** Descriptive physical characteristics (mean values ± standard deviations) of the participants (n = 37) in single remote ischemic preconditioning crossover experiment (Protocol – A).

Variable	Unit	Group I (n = 18)	Group II (n = 19)
Height	[cm]	180.31 ± 4.37	181.30 ± 4.77
Body mass	[kg]	75.15 ± 8.41	78.79 ± 8.66
Body Mass Index	[kg/m^2^]	23.11 ± 2.38	23.96 ± 2.37
Fat free mass	[kg]	37.74 ± 2.98	39.83 ± 3.91
Fat mass	[kg]	9.22 ± 5.22	9.45 ± 5.15
Percent body fat	[% body mass]	11.87 ± 5.11	11.72 ± 5.53

**TABLE 2:** Descriptive physical characteristics (mean values ± standard deviations) of participants (n = 37) in consecutive 10-day remote ischemic preconditioning (RIPC) training experiment (Protocol – B).

Variable	Unit	RIPC (n = 18)		SHAM (n = 19**)**	
		Before	After	Before	After
Height	[cm]	180.71 ± 3.19	-	180.84 ± 5.16	-
Body mass	[kg]	75.52 ± 7.51	75.01 ± 7.70	79.18 ± 8.99	79.03 ± 8.71
Body Mass Index	[kg/m^2^]	23.04 ± 2.21	22.95 ± 2.19	23.31 ± 2.29	24.19 ± 2.36
Fat free mass	[kg]	37.65 ± 2.26	37.56 ± 2.78	39.20 ± 3.97	39.48 ± 4.28
Fat mass	[kg]	9.77 ± 5.97	9.44 ± 5.65	10.75 ± 4.77	10.40 ± 4.58
Percent body fat	[% body mass]	12.48 ± 5.97	12.18 ± 5.92	13.33 ± 4.97	12.94 ± 5.06

### Procedures

#### Remote ischemic preconditioning

The procedure was performed in a supine position with unilateral arterial occlusion on a single, dominant lower limb. The arterial inflow was blocked using an occlusion cuff positioned proximally around the thigh and inflated to 220 mmHg (for the experimental population).

During the procedure, participants experienced four cycles of 5-minute cuff inflation followed by 5-minute deflation. The procedure was held in the morning hours (between 08:00 and 10:00 a.m.). Fifteen minutes before the start of the procedure and post the procedure participants remained in a supine position in order to normalize their cardiological parameters and well-being.

The efficiency of the arterial flow occlusion was monitored using Doppler ultrasonography (Edan DUS 60, Edan Instruments GmbH SonoTrax Basic, Langen, Germany). All ultrasound procedures adhered to the standards of the Polish Ultrasound Society and were performed by a physician trained in ultrasound imaging.

Participants that were assigned to SHAM group underwent a similar procedure (four cycles of 5-minute cuff inflation followed by 5-minute deflation in supine position with unilateral arterial occlusion on a single, dominant lower limb) but with an occlusion cuff inflated to only 20 mmHg, thus serving as a placebo.

#### Wingate anaerobic test

The WAnT specifically its 30-second version with full resistance applied to the flywheel from the onset, was utilized to assess the participants’ maximum anaerobic capacity as described by Bar-Or (Bar-Or, 1987). The test was conducted on a Monark Ergomedic 894E bicycle ergometer calibrated to a load of 0.075 kg per kilogram of body mass. The WAnT started with a 5-minute warm-up at 60 rpm against a 0.5-kg load followed by a 5-minute passive rest period. The maximum anaerobic capacity of the lower limbs was determined using the following indices: peak power (W), peak power normalized to body weight (W/kg), average power (W), average power normalized to body weight (W/kg), and fatigue index (%) expressed as the ratio of the work that was not accomplished to the work that would have been completed if the maximum power had been sustained until the end of the effort. Throughout the testing, all subjects received strong verbal encouragement from the test administrators to ensure their motivation during each trial.

#### Blood sample collection and analysis

To evaluate the effects of single and consecutive 10-day RIPC procedures on participants’ post-exercise inflammation markers, blood samples were collected. The blood samples were drawn into Monovette tubes (S-Monovette® Sarstedt AG&Co., Nümbrecht, Germany), with some containing coagulant for blood analyses and others without anticoagulant for serum separation (with a coagulation accelerator).

The serum was separated by centrifugation at 4,000 *g* for 10 min and aliquoted into 500 μL portions. The samples were frozen and stored (no longer than 6 months) at −80°C until further analysis. The MAGPIX fluorescence-based detection system (Luminex Corp., Austin, TX, United States) and Luminex assays (Luminex Corp.) were used for the assessment of the selected protein and inflammatory marker levels: interleukin 6 (IL-6), interleukin 10 (IL-10), interleukin 15 (IL-15), oncostatin M (OSM), leukemia inhibitory factor (LIF), and brain-derived neurotrophic factor (BDNF).

For glucose and lactate concentrations, fingertip capillary blood samples were collected. These samples were collected in plastic capillary tubes (20 µL) and injected into reagent-filled Safe-Lock Eppendorf tubes. The prepared blood samples were frozen at −80°C and analyzed using a Biosen C-Line glucose and lactate analyzer (EKF Diagnostics, Germany).

### Statistical analysis

Basic descriptive statistics were utilized to analyze the results. The normality of the data distribution was verified with the Shapiro-Wilk test. The differences in anthropometric characteristics between the two groups in protocol – A were assessed by an unpaired two-sample *t*-test. To analyze the effect of the single RIPC procedure on the participants’ maximal anaerobic capacity (in protocol – A), a one-way analysis of variance (ANOVA) with repeated measures was employed. For assessing the impact of the consecutive 10-day RIPC procedure on the participants’ maximal anaerobic performance, anthropometric characteristics and resting concentrations of inflammatory markers, a two-way ANOVA with repeated measures (2 × 2; group factor: RIPC, SHAM × time factor: before, after) was used. Additionally, another set of two-way ANOVA with repeated measures (2 × 3; group factor: RIPC, SHAM × time factor) was used to assess maximal anaerobic effort-induced changes in blood lactate, glucose (before, 3-, and 15-minutes post WAnT), and inflammatory marker levels (before, 60, and 120 min after WAnT) both after the single RIPC as well as before and after the consecutive 10-day RIPC interventions.

To determine the effect size, the Eta-squared statistics (η^2^) was used, where values equal to or exceeding 0.01, 0.06, and 0.14 indicated a small, moderate, and large effect, accordingly. In the case of a significant effect of the main factor or factor interaction in the repeated measures analyses of variance, Tukey’s *post hoc* test was used.

The statistical significance of all performed tests was determined at the level of α = 0.05. Statistical analysis was performed using Statistica 12 software (StatSoft Inc. Tulsa, OK, United States).

## Results

### Protocol - A. single RIPC intervention

No differences in anthropometric characteristics between Group I and II were observed.

Wingate Anaerobic test results after a single RIPC procedure are shown in Table 3. One-way ANOVA with repeated measures showed no significant differences between the group in WAnT performance after single RIPC procedure.

**TABLE 3:** Wingate Anaerobic Test results after the single remote ischemic preconditioning (RIPC) crossover experiment (n = 37) (Protocol – A) (mean values ± standard deviations).

Variable	Unit	RIPC (n = 18)	SHAM (n = 19)
Absolute peak power	[W]	755.20 ± 89.19	765.83 ± 127.10
Absolute mean power	[W]	589.54 ± 74.19	597.30 ± 91.97
Relative peak power	[W/kg]	10.01 ± 0.73	9.69 ± 0.86
Relative mean power	[W/kg]	7.87 ± 0.56	7.57 ± 0.62

**FIGURE 2:**
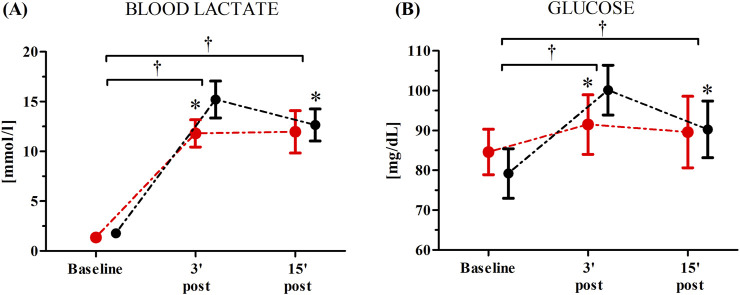
Blood lactate **(A)** and glucose **(B)** concentrations after the single remote ischemic preconditioning (RIPC) procedure as induced by Wingate Anaerobic Test (WAnT) (reported as mean values and standard deviations). Black – group receiving RIPC intervention; red – group receiving SHAM intervention. *significant difference with RIPC at 3 min post WAnT; at p < 0.01; † significant effect of time factor at p < 0.01.

Changes in the participants’ blood lactate and glucose concentrations as induced by the WAnT after the single RIPC procedure are shown in Figure 2.

An ANOVA showed a significant effect of time factor of blood lactate (F = 787.38, p < 0.01, η^2^ = 0.96) and glucose (F = 43.21, p < 0.01, η^2^ = 0.59) levels as induced by the WAnT after the single RIPC procedure. Regardless of group, a significant increase in the participants’ concentration of blood lactate was observed 3 min post (762.8%, p < 0.01) and 15 min post the WAnT (709.9%, p < 0.01) compared to the baseline values. Similarly, there was a significant increase in the blood glucose levels 3 min post (15.7%, p < 0.01) and 15 min post the WAnT (9.6%, p < 0.01). A significant group × time factors interaction was found for both blood lactate (F = 10.22, p < 0.01, η^2^ = 0.24) and blood glucose (F = 10.54, p < 0.01, η^2^ = 0.26). Post-hoc comparisons revealed significantly higher blood lactate (22.80%, p < 0.05) and blood glucose (8.98%, p < 0.05) levels 3 min post-WAnT in the RIPC group compared to the SHAM group (Figure 2).

Post hoc analysis also revealed a significant decrease in the blood lactate (−15.1%, p < 0.05) and blood glucose (−9.8%, p < 0.05) levels when comparing 15 min post-WAnT with 3 min post–WAnT in the RIPC group. In the SHAM group, the blood lactate and glucose concentrations recorded 3 and 15 min post the maximum anaerobic exercise was at a similar level.

**FIGURE 3:**
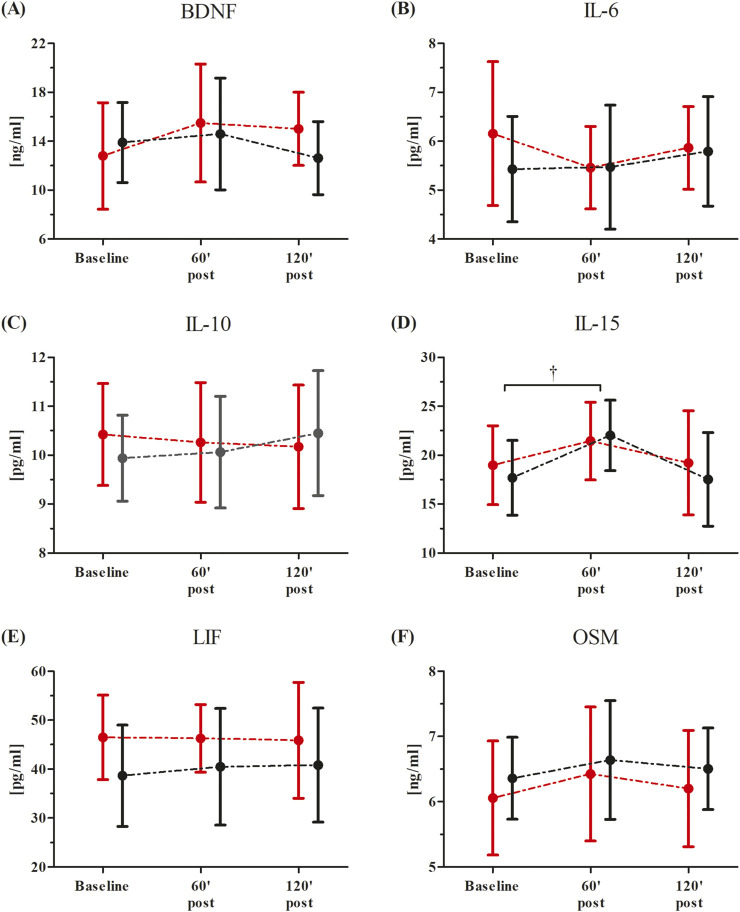
Changes in serum inflammatory markers after the single Remote Ischemic Preconditioning (RIPC) procedure as induced by the Wingate Anaerobic Test (WAnT) (reported as mean values and standard deviations). Black – group receiving single RIPC intervention; red – group receiving SHAM intervention. Abbreviations: **(A)** BDNF, Brain Derived Neurotrophic Factor; **(B)** IL-6, interleukin 6; **(C, B)** IL-10, interleukin 10; **(D)** IL-15, interleukin 15; **(E)** LIF, leukemia inhibitory factor; **(F)** OSM, oncostatin M; † significant effect of time factor at p < 0.01.

Changes in participants’ serum BDNF and inflammatory markers after the single RIPC as induced by the WAnT are shown in Figure 3. An ANOVA showed a significant effect of the time factor in IL-15 (F = 4.65, p < 0.05, η^2^ = 0.18). Regardless of the group division, a significant increase in IL-15 concentration was observed 60 min post-WAnT (18.0%, p < 0.05). No other effects were observed.

### Protocol - B. consecutive 10-day RIPC intervention

There were no significant differences in anthropometric characteristics between RIPC and SHAM groups, and also no significant changes within each group after consecutive 10-day of RIPC or SHAM interventions.

The ANOVA showed no significant changes in the resting levels of BDNF and inflammatory markers after consecutive 10 days of RIPC.

The WAnT results from before and after the consecutive 10-day RIPC procedure are detailed in Table 4. A significant group × time factors interaction for both the absolute peak (F = 5.38, p < 0.05, η^2^ = 0.13) and mean power (F = 6.28, p < 0.05, η^2^ = 0.15) as well as for the relative peak power (F = 5.86, p < 0.05, η^2^ = 0.14) and mean power (F = 6.53, p < 0.05, η^2^ = 0.16) was found.

**TABLE 4:** Wingate anaerobic test results before and after the consecutive 10-day remote ischemic preconditioning (RIPC) training experiment (Protocol – B) (mean values ± standard deviations).

Variable	Unit	RIPC (n = 18)		SHAM (n = 19)	
		Before	After	Before	After
Absolute peak power	[W]	703.66 ± 89.01	747.44 ± 84.96*	766.51 ± 130.54	778.46 ± 121.06
Absolute mean power	[W]	559.99 ± 71.10	586.83 ± 70.44*	581.93 ± 91.64	590.07 ± 87.71
Relative peak power	[W/kg]	9.41 ± 0.79	10.20 ± 0.66*	9.57 ± 0.88	9.70 ± 0.87
Relative mean power	[W/kg]	7.49 ± 0.67	7.84 ± 0.58*	7.50 ± 0.57	7.58 ± 0.59

Note: *significant difference vs. before in RIPC, group at p < 0.05.

Post-hoc analysis showed a significant increase in the absolute peak (6.25%, p < 0.05) and mean power (4.81%, p < 0.05) as well as the relative peak (6.38%, p < 0.05) and mean power (4.79%, p < 0.05) in RIPC group. In the SHAM group, the WAnT results showed no significant changes before and after the experiment.

**FIGURE 4:**
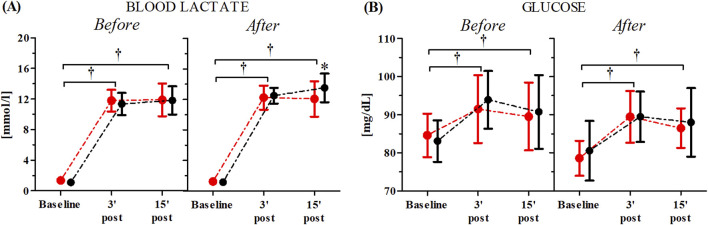
Changes in blood lactate **(A)** and glucose **(B)** concentrations after the consecutive 10-day RIPC procedure as induced by WAnT (reported as mean values and standard deviations). Black–group receiving single RIPC intervention; red – group receiving SHAM intervention. *significant difference between group at p < 0.01; † significant effect of time factor at p < 0.01.

The changes in participants’ blood lactate and glucose concentrations both before and after the consecutive 10-day RIPC procedure as induced by the WAnT are shown in Figure 4. The ANOVA for the blood lactate levels demonstrated a significant time factor effect both before (F = 1,357.98, p < 0.01, η^2^ = 0.96) and after (F = 1,601.29, p < 0.01, η^2^ = 0.97) the consecutive 10-day RIPC procedure. Similarly, significant effects were observed for the blood glucose levels before (F = 24.77, p < 0.01, η^2^ = 0.44) and after (F = 27.21, p < 0.01, η^2^ = 0.45) the RIPC procedure. A significant group × time factors interaction was noted only for the blood lactate levels (F = 4.54, p < 0.01, η^2^ = 0.11). Post-hoc analysis showed a significantly higher blood lactate concentration (12.08%, p < 0.05) 15-minutes post-WAnT in the RIPC group compared to the SHAM group (Figure 4).

**FIGURE 5:**
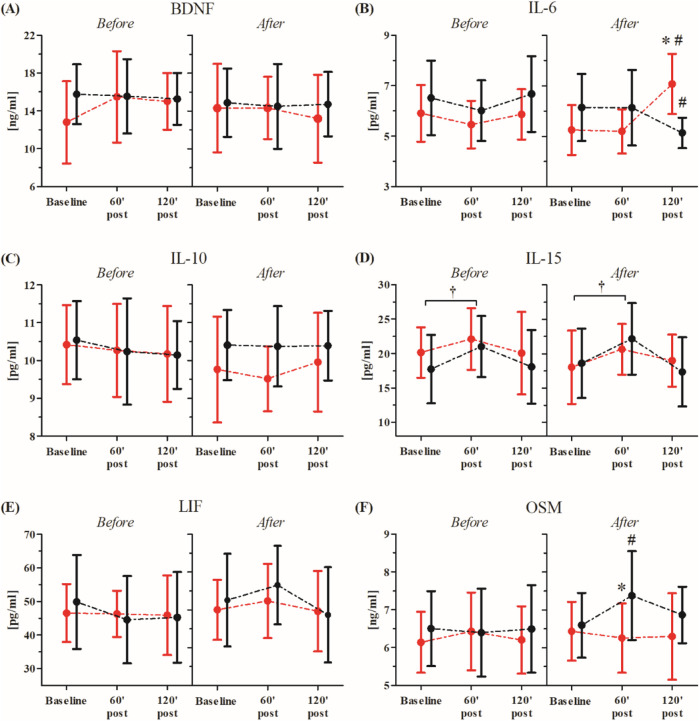
Changes in serum BDNF and inflammatory markers before and after consecutive 10-day RIPC procedure as induced by WAnT (reported as mean values and standard deviations). Black–group receiving consecutive 10-day RIPC procedure; red – group receiving SHAM intervention (SHAM). Abbreviations: **(A)** BDNF, brain derived neurotrophic factor; **(B)** IL-6, interleukin 6 **(C, B)** IL-10, interleukin 10; **(D)** IL-15, interleukin 15; **(E)** LIF, leukemia inhibitory factor; **(F)** OSM, oncostatin M. *significant difference between groups at particular sampling time point at p < 0.01; #significant difference with baseline value in particular group at p < 0.01; † significant effect of time factor at p < 0.01.

The WAnT-induced changes in participants’ serum inflammatory markers before and after the consecutive 10-day RIPC procedure are shown in Figure 5.

The ANOVA before and after the consecutive 10-day RIPC procedure showed a significant effect of the time factor for only IL-15 (before, F = 3.25, p < 0.05, η^2^ = 0.13; after, F = 3.64, p < 0.05, η^2^ = 0.14). Regardless of the group division, both before (13.9%, p < 0.05) and after the intervention (16.8%, p < 0.05), a significant increase in the IL-15 concentration in comparison to the baseline values was observed 60 min post the WAnT.

A significant group × time factors interaction of the tested inflammatory markers was only noted in IL-6 (F = 9.93, p < 0.01, η^2^ = 0.29) and OSM (F = 3.11, p < 0.05, η^2^ = 0.13).

Post-hoc comparisons revealed a significant decrease in the IL-6 (15.5%, p < 0.05) concentration at 120 min post-WAnT in the RIPC group compared to the baseline. On the other hand, in the SHAM group, a significant increase in IL-6 (20.6%, p < 0.01) concentration was observed between the same time points. Post-hoc analysis also showed a significantly higher concentration of IL-6 (23.6%, p < 0.01) in the SHAM group at 120 min post-WAnT.

In the case of OSM, a significant increase in concentration 60 min post-WAnT was observed in the RIPC group (12.2%, p < 0.01), while, in the SHAM group, no significant changes between the selected time points were noted.

## Discussion

In the present research, we demonstrated that a consecutive 10-day RIPC procedure applied on a single leg significantly enhanced sports performance. Moreover, the effects of single and consecutive 10-day RIPC procedures on selected inflammatory markers and myokine secretion induced by maximal anaerobic effort were evaluated. To the best of our knowledge, this is the first study where the effectiveness of a single *versus* consecutive 10-day RIPC procedure has been compared. Here, we observed an increase in participants’ WAnT mean and peak power after the consecutive 10-day RIPC, while the single RIPC was found to have no such effects. These data confirmed previous observations in which a consecutive 10-day RIPC procedure on both arms led to a significant improvement in the average relative power (from 5.29 into 5.79 W/kg) during an upper-limb WAnT (Mieszkowski et al., 2019). The lack of effect of a single RIPC on performance aligns with the results of Lalonde and Curnier, who showed that RIPC does not offer any significant benefits for anaerobic performance (Lalonde and Curnier, 2015). A similar conclusion was reached by Paixao et al. (2014), who showed that a single RIPC procedure may even lead to a deterioration in maximal anaerobic performance due to the fatigue of the affected tissue. Conversely, it has been shown that the RIPC procedure effectively induces some positive changes in performance and erythrocyte deformability in some but not all subjects (Tomschi et al., 2018).

Physical performance during 30 s WAnT is mainly based on glycolytic ATP production (de Poli et al., 2021); thus, lactate and glucose were measured after the exercise. Single RIPC procedures modulated post-exercise glucose and lactate concentrations. It is interesting to note that significantly higher values of both lactate and glucose were observed in the capillary blood 3 min after the WAnT in the RIPC group, which suggests that despite having no effects on performance, RIPC induced some metabolic changes. Higher lactate formation during this kind of exercise should be considered a positive change, indicating a faster rate of glycolysis. Conversely, a significant decrease in lactate and glucose concentrations between the 3rd and 15th minute in the RIPC group may additionally point to participants’ better recovery after the exercise. Lactate, after an exercise, can be a substrate for liver gluconeogenesis or can be taken up by skeletal muscle and the brain, where it can be oxidized (Matsui et al., 2011; Bailey et al., 2012; Thom et al., 2020). Lactate has also been shown to stimulate IL-6 release from skeletal muscle (Hojman et al., 2019). Thus, it can be expected that higher serum lactate will be associated with serum IL-6 but this is not the case. As IL-6 can stimulate liver glycogenolysis, which can lead to increased serum glucose, again interdependence can be expected (Barnes et al., 2014). Here, we observed higher serum glucose after RIPC but no correlation with IL-6.

Conversely, in subjects after the 10-day RIPC procedure, a decrease in IL-6 and an increase in the SHAM group were observed 2 h after the exercise, but the changes in glucose were the same in both groups. Previous research has shown that exercise-dependent IL-6 levels may also be mediated by metalloproteinases (MMP2 and MMPx); inactivation of these proteins blunts IL-6 release induced by swimming (Hojman et al., 2019). Previously, on marathon runners, it was demonstrated that the levels of TIMP- 1, a tissue inhibitor of metalloproteinases, significantly decreased after the marathon, and this effect was attenuated in runners after RIPC (Mieszkowski et al., 2020). Thus, it is possible that RIPC training can influence IL-6 release by modulating the activity of metalloproteinases.

It is worth noting that several authors have not observed significant effects of RIPC on exercise-induced changes in lactate concentration (de Groot et al., 2010; Crisafulli et al., 2011; Jean-St-Michel et al., 2011; Bailey et al., 2012; Clevidence et al., 2012; Paixão et al., 2014). Such a situation may result from different experimental conditions; for example, none of these studies used the WAnT, an exercise mainly based on anaerobic ATP resynthesis systems. In summarising the results of the research by other authors and that presented in this work, it can be concluded that a single RIPC procedure may not be a sufficient stimulus to induce beneficial changes in a person’s maximum anaerobic capacity despite its effects on exercise metabolism.

Recent studies by Mieszkowski et al. (2020), Mieszkowski et al. (2021) on the impact of lower-limb RIPC procedures on biochemical changes induced by physical activity have proven the potential benefits of using this specific training method in modulating post-exercise serum levels of selected inflammation markers proved that a consecutive 10-day RIPC procedure performed on lower limbs contributes to positive biochemical adaptive changes (a decrease in inflammation marker secretion and increased muscle post-exercise stress resistance) as induced by long-distance running. Moreover, Mieszkowski et al. (2021) additionally proved that a consecutive 10-day RIPC procedure generates a protective effect by decreasing post-exercise liver and heart injury serum markers and reducing oxidative stress as induced by a marathon run.

In the current study, we also aimed to evaluate how RIPC performed on a single dominant leg would modulate exerkine levels and inflammation markers during maximal anaerobic testing. There is very limited data on the effect of the WAnT on myokine concentrations (Son et al., 2018). Therefore, in the study, we investigated the effect of the WAnT exercises and RIPC procedures on the concentration of BDNF, IL-6, IL-10, OSM, LIF, and IL-15. It was shown that only a consecutive 10-day RIPC procedure was able to change some of the exerkine response after WAnT in comparison with SHAM, while a single RIPC was ineffective. It was shown that only a consecutive 10-day RIPC procedure was able to change exercise response after WAnT, specifically IL-6 and OSM. OSM is classified within the IL-6 group of cytokines, which was found to stimulate the inflammatory response in cell cultures and affect cell growth (Yao et al., 1996; Ouyang et al., 2006; Hojman et al., 2011; Pan et al., 2016). An increase in OSM after the WAnT was observed in the RIPC trained. This observation is consistent with previous studies showing that its synthesis and release into the serum are stimulated by exercise (Hojman et al., 2011). Conversely, neither marathon runs nor combined with RIPC training before affected OSM (Mieszkowski et al., 2020). Thus, possibly, that type of exercise and its intensity may have influenced the synthesis of this cytokine. In the case of IL-6, RIPC training attenuated this increase, observed 120 min after the exercise, consistent with a previously published study on marathon runners (Mieszkowski et al., 2020).

Thus, observed changes induced by consecutive 10-day RIPC and exercise could attract broader interest beyond that of sports physiologists or biochemists. Further studies are required to fully understand the molecular mechanism(s) that underpin the effect of ischemic preconditioning on exercise-induced inflammation.

In conclusion, we showed that a consecutive 10-day RIPC procedure, in contrast to a single RIPC, increased the maximal anaerobic performance with the glucose and lactate positive changes. This was associated with reduced post-exercise IL-6 concentration and modulated OSM levels. This suggests that repeated RIPC procedures could be an alternative and additional training method. Moreover, changes in post-exercise response suggest additional metabolic adaptation that could contribute to better exercise tolerance.

## Limitation

Despite the observed beneficial effects of the presented research protocol on the post-exercise response, the interpretation of the obtained results is associated with certain limitations. The SHAM procedure used in the control population may not fully meet the control criteria in studies, as is the case when using two different, e.g., supplementation protocols. Using four cycles of 5-minute cuff inflation to 20 mmHg followed by 5-minute deflation certainly causes different sensations in the test subjects–and may not be associated with ex. numbness, soreness or tingling. However, the necessity of occlusion of large arteries in the RIPC procedure does not allow for any other method of control intervention. Another issue that should be pointed out is that the same one lower limb received intervention and then was used along with other one to perform WAnT, which could not be perceived as RIPC, but more as IPC. It was previously proven that despite local influence of IPC its effects are extended on whole body functioning (Mieszkowski et al., 2021). During strenuous exercise inflammation process occurs not only in working muscles but with the blood stream it spreads on whole body system (Peake et al., 2005). It was observed by Mieszkowski et al. (2021) in marathon runners that ischemic preconditioning of lower limbs reduces marathon-induced oxidative stress and decreases liver and heart injury markers in the serum (as a distant effect). Moreover, it was also previously shown that the ischemic preconditioning on one lower limb can influence the neuromuscular performance of the contralateral limb (Niespodziński et al., 2021). Bearing in mind the above, the aim of the study, and the fact that systemic blood samples were analyzed instead of muscle tissue itself, we believe that using RIPC term in current study is justified. Moreover, the effectivity of cuff pressure during the experiment could be additionally measured with pulse oximetry. This type of measurement could show how performed RIPC and SHAM models affect blood oxygenation, so this type of measurement should be included in the study protocol in future research.

Moreover, during the presented studies, it would be interesting to compare single, dominant lower limb intervention with not-dominant limbs and both limbs’ ischemic preconditioning procedures with different duration of the ischemia (days of ischemic preconditioning training).

However, we suggest that the main mechanism of ischemic preconditioning activity is not connected to the limb dominant function but with the biochemical adaptations, and observations on both legs could show much more pronounced changes but with very similar directions.

## Data Availability

The raw data supporting the conclusions of this article will be made available by the authors, without undue reservation.

## Abbreviations

RIPC, remote ischemic preconditioning; IL-6, interleukin 6; IL-10, interleukin 10; IL-15, interleukin 15; OSM, oncostatin M; LIF, leukemia inhibitory factor; BDNF, brain-derived neurotrophic factor; WAnT, Wingate Anaerobic Test.
