# Supplementary material for: Single and consecutive 10-day remote ischemic preconditioning modify physical performance, post-exercise exerkine levels, and inflammation
Source: Front Physiol. 2024 Nov 11;15:1428404. doi: 10.3389/fphys.2024.1428404 (PMC11586381; doi:10.3389/fphys.2024.1428404)
Supplement: Supplementary file 1 [file Table1.docx]

**Table S1.** Descriptive physical characteristics of participants (n = 37) in consecutive 10-day remote ischemic preconditioning (RIPC) training experiment (Protocol – B)

| Variable | Unit | | RIPC  (n = 18) | | | SHAM  (n = 19) | | | |
| --- | --- | --- | --- | --- | --- | --- | --- | --- | --- |
|  |  |  | Before | After | P | | Before | After | P |
| Height | | [cm] | 180.71 ± 3.19 | - | - | | 180.84 ± 5.16 |  |  |
| Body mass | | [kg] | 75.52 ± 7.51 | 75.01 ± 7.70 | 0.53 | | 79.18 ± 8.99 | 79.03 ± 8.71 | 0.89 |
| Body Mass Index | | [kg/m²] | 23.04 ± 2.21 | 22.95 ± 2.19 | 0.48 | | 23.31 ± 2.29 | 24.19 ± 2.36 | 0.81 |
| Fat free mass | | [kg] | 37.65 ± 2.26 | 37.56 ± 2.78 | 0.90 | | 39.20 ± 3.97 | 39.48 ± 4.28 | 0.87 |
| Fat mass | | [kg] | 9.77 ± 5.97 | 9.44 ± 5.65 | 0.28 | | 10.75 ± 4.77 | 10.40 ± 4.58 | 0.21 |
| Percent body fat | | [%body mass] | 12.48 ± 5.97 | 12.18 ± 5.92 | 0.42 | | 13.33 ± 4.97 | 12.94 ± 5.06 | 0.16 |
